# The Process of Spiritual Care

**DOI:** 10.3389/fpsyg.2021.674453

**Published:** 2021-09-07

**Authors:** Ricko Damberg Nissen, Dorte Toudal Viftrup, Niels Christian Hvidt

**Affiliations:** Research Unit of General Practice, Department of Public Health, University of Southern Denmark, Odense, Denmark

**Keywords:** spiritual care, meaning-making, ontological grounding, secular, spiritual, religious

## Abstract

The aim of this article is to illustrate and outline an understanding of spiritual care as a process involving a number of organically linked phases: (1) the identification of spiritual needs and resources, (2) understanding the patient’s specific needs, (3) developing the individual spiritual care treatment plan, hereunder involving the relevant healthcare/spiritual care professionals, (4) the provision of spiritual care, and (5) evaluating the spiritual care provided. The focus on spiritual care in healthcare research has increased throughout the past decades, showing that existential, spiritual, and/or religious considerations and needs increase with life-threatening illness, that these needs intensify with the severity of disease and with the prospect of death. Furthermore, research has shown that spiritual care increases quality of life, but also that failing to provide spiritual care leads to increased chance of depression and lowered health conditions. The World Health Organization accordingly emphasizes that providing spiritual care is vital for enhancing quality-of-life. Looking at spiritual care as a process suggests that working within a defined conceptual framework for providing spiritual care, is a recommendable default position for any institution where spiritual care is part of the daily work and routines. This so, especially because looking at spiritual care as a process highlights that moving from identifying spiritual needs in a patient to the actual provision of spiritual care, involves deliberate and considered actions and interventions that take into account the specific cultural and ontological grounding of the patient as well as the appropriate persons to provide the spiritual care. By presenting spiritual care as a process, we hope to inspire and to contribute to the international development of spiritual care, by enabling sharing experiences and best-practices internationally and cross-culturally. This so to better approach the practical and daily dimensions of spiritual care, to better address and consider the individual patient’s specific spiritual needs, be they secular, spiritual and/or religious. In the final instance, spiritual care has only one ambition; to help the individual human being through crisis.

## Introduction

Spiritual care is an important aspect of patient centered care and in healthcare research the focus on spiritual care has been growing through the past decades (Cadge and Bandini, 2015; Gijsberts et al., 2019; Harrad et al., 2019). In Geriatrics, for instance, research has found that spirituality and religion is supportive of health and well-being in old age (Rykkje et al., 2013) and that older people going through illness or approaching death are in high risk of experiencing a spiritual crisis (Wiltjer, 2019). Research has also shown, however, that spiritual needs are often overlooked in healthcare in general and that spiritual care is difficult to integrate as part of daily care and disease management (Assing Hvidt et al., 2017a; Straßner et al., 2019). The World Health Organization thus emphasizes that providing spiritual care is vital for enhancing quality-of-life and should be included in treatment (Group, 1994). The Joint Commission on Accreditation of Healthcare Organizations (JCAHO) in the United States has stipulated that spiritual care should be included in medical and nursing education (Hodge, 2006), just as countries in Europe have included spiritual care in the curriculum in medical training (Taverna et al., 2019; Viftrup et al., 2021).

Providing spiritual care in a global, culturally entwined, and pluralistic world (Taylor, 2007; Berger, 2014) is complicated, as providers have to be sensitive to the potential variance in the secular, spiritual, and religious meaning orientations of their patients (Ghorbani et al., 2021). A wide range of interventions have been developed in the area of spiritual care, but research shows that these interventions are often developed as stand-alone instruments, such as for instance questionnaires or interview guides assessing spiritual needs (Damberg Nissen et al., 2020), and do not approach spiritual care as an ongoing and integrated part of patientcare. One of the reasons for this is that spiritual needs are always individual, and how spiritual care is provided depends on these specific needs, but also on the relationship between patient and provider(s) (Moudatsou et al., 2020). Therefore, it often falls on the individual healthcare professional (HCP) to incorporate spiritual care in day-to-day care, and combined with ethical and cultural considerations, professional and personal boundaries, lack of time and resources, and so on, it is understandable that HCPs often find it challenging to include spiritual care in daily care (Assing Hvidt et al., 2017a; Damberg Nissen et al., 2018; Teófilo et al., 2019; Moudatsou et al., 2020). This leads to a situation where the provision of spiritual care is in risk of becoming an auto-didact and *ad hoc* solution, arbitrarily implemented, or marginalized altogether (Hvidt et al., 2016; Austin et al., 2018). As an approach to address and overcome these difficulties, we argue that spiritual care should be regarded as a process.

The aim of this article is to illustrate and outline an understanding of spiritual care as a process involving a number of organically linked phases: (1) the identification of spiritual needs and resources, (2) understanding the patient’s specific needs, (3) developing an individual spiritual care treatment plan (hereunder involving the relevant healthcare/spiritual care professionals), (4) the provision of spiritual care, and (5) evaluating the spiritual care provided.

Every local context, patient, and provision of spiritual care is unique, and we need to appreciate this. However, we also need to appreciate the need for international research and exchange of knowledge and experience, to further the development of spiritual care. Therefore, the aim is not to present a spiritual care intervention *per se*, but an understanding of spiritual care as a structured process, that will enable both the local development and provision of spiritual care and the international exchange of practice and experience.

Positing spiritual care as a process brings to attention that spiritual care instruments and interventions are often focused on specific aspects of spiritual care, but also that combining existing instruments in a spiritual care treatment plan is possible and can be a beneficial approach to providing spiritual care (Damberg Nissen et al., 2020; Nissen and Hvidt, 2021). It also brings to attention that work is needed in the area of developing and training for providing spiritual care. This work is already commencing, as reflected in the work of the WHO and JCAHO mentioned above, but also in spiritual care training programs, such as the “Interprofessional Spiritual Care Education Curriculum,” developed at the George Washington School of Medicine & Health Sciences (Bandini et al., 2018; Puchalski et al., 2020).

In this article, the primary healthcare area is Geriatrics, old age/late life. However, spiritual care is part of many healthcare areas and as such, looking at spiritual care as a process is relevant for any healthcare area where spiritual care is part of daily practice, such as palliative care (Gijsberts et al., 2019), oncology (Conway, 2010), orthopedic (Clark, 1997), or around childbirth (Crowther and Hall, 2015). It should also be mentioned that understanding patientcare as a process in general is well-established and includes processes similar to what we suggest here, such as assessment, development of care plan, and provision of and follow-up on such care (Bergman et al., 2011; T. L. C., 2019). Nevertheless, research has shown that when it comes to spiritual care, the provision of spiritual care is often arbitrary, auto-didact, and linked to personal values (Hvidt et al., 2016; Austin et al., 2018).

Spiritual care is further complicated by the concept “spiritual,” which has (so far) defied unified international definitions, just as the relation between spirituality and health needs to be better understood (Delgado, 2005; Hvidt et al., 2021). A recent study from Germany concludes that the concept “spirituality” is immature in the German language (Grabenweger and Paal, 2021). This illustrates how international understandings and discussions of spirituality and spiritual care can be difficult to incorporate in local vernacular understandings of how to approach the patient in relation to the individual patient’s spiritual needs and resources (Daaleman, 2012; Caldeira et al., 2013).

As a way to address this and the complexity of a culturally entwined and pluralistic world, we introduce the concept “ontological grounding,” inspired partly by Giddens (2018) concept “ontological security” and partly by Holbraad and Madsen’s “ontological turn” (Holbraad, 2017). Through this, we hope to contribute with an understanding of spiritual care that will enable clinical practice, palliative care, oncology, geriatrics, and other healthcare areas, to approach spiritual care in a systematic way, sensitive to the secular and pluralistic character of a culturally entwined world, and thereby also to contribute to the continuing international development of spiritual care.

In our conceptualization of spiritual care, we focus on secular, spiritual and religious existential orientations, needs, and resources in connection with illness and crisis. This understanding is aimed at capturing the potential width of the individual patient in relation to the ontological grounding (la Cour and Hvidt, 2010; Nolan, 2011; Hvidt et al., 2020, 2021).

In the following, we outline the process of spiritual care as a local undertaking in a culturally entwined and pluralistic world. We introduce “the ontological grounding” and “the Meaning-Making Matrix,” as a way to approach the individual patient, sensitive to the individual patient’s specific secular/spiritual/religious, and cultural existential orientation (la Cour and Hvidt, 2010; Nissen, 2019). Hereafter, we outline and discuss the process of spiritual care, exemplified by instruments working in the different phases of the process. The examples in the discussion are drawn from the Catalogue of Spiritual Care Instruments (Damberg Nissen et al., 2020).

## The Ontological Grounding and the Meaning-Making Matrix

A central aspect in the following understanding and discussion of spiritual care as a process is that we entertain an understanding of the world as culturally entwined and pluralistic (Taylor, 2007; Mignolo, 2011; Berger, 2014), in the sense that secular, spiritual, and religious people live entwined in a surrounding cultural context; they are neighbors, they are colleagues, they commute, and sit next to each other on the bus and the train, and… they use the same healthcare systems. However, they do not necessarily know anything about each other. They don’t know whether the person sitting next to them on the bus is happily married, just got promoted, or is in deep existential crisis, because of a tumor just being diagnosed as terminal. The physician, the nurse, the chaplain, the relative, the friend, or whoever this person may be on the way to for counsel and help, do not have access to the innermost thoughts and feelings of this person, not for all the empathy in the world. However, and despite this level of separateness from each other, we are also connected through our shared humanity, offering us the social contexts that influence who we are, giving us ontological security (Giddens, 2018), enabling the empathy we need to understand and support each other, indeed for providing spiritual care. This is what we argue as the ontological grounding of the individual, the parts of the individual that others do not have access to and cannot know, and the empathetic ability of us to understand each other despite these limitations.

In a culturally entwined and pluralistic world, an existential orientation, be it secular, spiritual, or religious, is a conscious choice for some and not so for others (Taylor, 2007). For some it is irrelevant, for others it is the Archimedean point around which everything else revolves (Damberg Nissen et al., 2018). However, when faced with life-threatening illness these existential questions have a strong tendency to surface (Hvidt et al., 2017, 2019). A religious person may draw from his/her religiosity the mental, physical, or social strength to cope with a life-threatening diagnose, but the same religiosity can also lead to a complete collapse in meaning, followed by doubt, guilt, anger, depression, even suicide. Similarly, a secular person faced with a life-threatening diagnosis may draw from this secular orientation the strength to either cope with the situation or experience a collapse in meaning and find him or herself on the brink of depression or worse (Moestrup and Hvidt, 2016; Hvidt et al., 2017). If spiritual care is to be patient centered and patient empowering, it is necessary to ascertain an understanding of the patient’s ontological grounding, in order to understand from which aspects (secular, spiritual, religious) and to what degree the meaning-making process is stable or has collapsed. This also calls for an appreciation of whether the needs are of a cognitive or practical kind, to differentiate and plan the spiritual care accordingly.

In order to empower local understandings and to enable cross-cultural exchange of knowledge and experience, it is pivotal to engage the three central concepts; secular, spiritual, religious, as Western constructs with varied meaning, connotations, and importance in different contexts, international, local vernacular, and individual levels (Mignolo, 2011; Bowman, 2014). This shows us the limits of the concepts we use and the difficulties of using them cross-culturally, where especially the concept “spiritual” lacks international consensus, definition, and even usability, which again makes it even harder to define what spiritual care is. It is depending on the local context. Following this, it might seem artificial and even counterproductive to separate human meaning-making processes into the existential domains secular, spiritual, religious, even more so as patients may think about existence in secular, spiritual, religious terms simultaneously, or move between them and place different importance in them at different times (Berger, 2014; Johannesen-Henry CaI, 2019). Thus, the domains may be entwined (la Cour and Hvidt, 2010). These are the concepts we have; these are our limitations. However, precisely by engaging these concepts actively we highlight that the human worlds we try to capture through these concepts and models do not easily render themselves to such constructs (Descola, 2014; Holbraad, 2017), which is an important aspect, when attempting to approach a patient’s ontological grounding.

By attempting to reach an understanding of a patient’s ontological grounding, we highlight that even though some things lie outside of our reach, this does not mean that we should not attempt to reach an understanding and therethrough an appreciation of them. To some extent, this will enable us to transgress the concepts secular, spiritual, religious, and therethrough better identify the character of the identified spiritual needs and to develop the appropriate approach to providing spiritual care.

**FIGURE 1:**
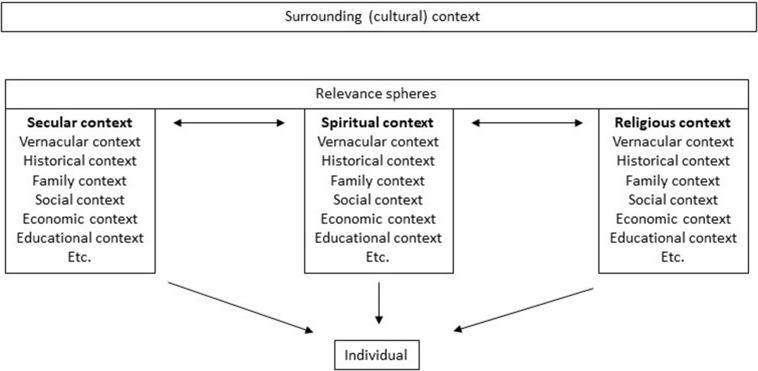
The ontological grounding.

Figure 1 illustrates the ontological grounding of the individual differentiated in three spheres of relevance (Berger, 2016): secular, spiritual, and religious. The “Surrounding (cultural) context” signifies the context in which the individual lives, such as for instance Canada, Denmark, Germany, etc. The underlying “Relevance Spheres” and “Secular context,” “Spiritual context,” “Religious context,” mention some of the significant influencers on the ontological grounding of the individual. These may differ from the surrounding (cultural) context in the sense that the surrounding (cultural) context is biased, promoting certain things while discouraging others; there are no value free settings (Kørup et al., 2020). In a healthcare context this could for example be promoting exercise while discouraging smoking. The relevance spheres may or may not agree with this, and the individual “makes up his/her own mind,” so to speak. As illustrated by the identical content of the three boxes individuals are naturally influenced by similar influencers that comes from learning a specific language, living in a specific family with a specific history and economy and so on, while also highlighting that even though these influencers are similar, the individuals are still unique. The arrows between the boxes illustrate that an individual is likely to move between the spheres depending on context. As Peter Berger argued, people most often have no problem with moving between relevance spheres and can quite easily differentiate between when it is time to be (act) secular and when it is time to be (act) religious (Berger, 2014, 2015). However, in the context of secular healthcare this might not be so easy, as the patient consulting the physician (in a secular setting) is informed by the surrounding cultural context that this is not the time, nor the place, to talk about spiritual pains and needs, and thereby the patient may marginalize his/her spiritual needs, and resources for that matter. As research has shown, the HCP may also find it difficult and sometimes even irrelevant to bring up spiritual needs, and thereby also, maybe unintentionally, marginalize the patient’s spiritual needs (Nissen et al., 2019a). With the words of Charles Taylor, it can be argued that the existential orientations in contemporary Western culture, be they secular, spiritual or religious, are cross-pressured in a figurative force-field, they are continuously contested by the presence of each other and work upon each other as interior “pressures” or “forces,” thereby fragilizing each other (Taylor, 2007). For healthcare, this may explain why it is difficult to approach existential orientations due to the presence of barrier pressures (the bias of the surrounding (cultural) context) of, for instance, ethics, professional boundaries, and scientific discourse, that are countered by facilitating pressures of, for instance, compassion and sense of a patients’ spiritual needs (Damberg Nissen et al., 2018). These types of barriers and facilitators work against each other in the clinic and constitute the force field of opposing cross-pressures (barriers and facilitators) that HCPs need to engage in the encounter with each new patient.

Figure 1 thus illustrates the surrounding (cultural) context and relevance spheres as significant influencers on the ontological grounding of the individual, yet every individual is physically and mentally separated from everyone else. We are semantically situated in relation to each other through the surrounding (cultural) context and relevance spheres and therefore we have similar understandings of the world, but we are not the same. We do not have access to the “inner life” of each other. In the final instance, the ontological grounding of the individual is unique.

We argue that an attempt at understanding of the ontological grounding of the individual patient is necessary, even imperative, for providing spiritual care in a culturally entwined and pluralistic world. How to approach the patient and provide spiritual care from this understanding is not easily put into formulae or practice. However, being explicit and proactive about it and approaching an understanding of the patient from this perspective, will enable more explicit and inclusive reflections in relation to the individual, and help the HCP gage from which perspectives and to what degree the existential meaning-making of the individual patient has collapsed. From here the HCP will be in a better position to develop a spiritual care treatment plan that is sensitive to the ontological grounding, appropriate, inclusive, empowering, and, not the least, recognizable to the patient.

**FIGURE 2:**
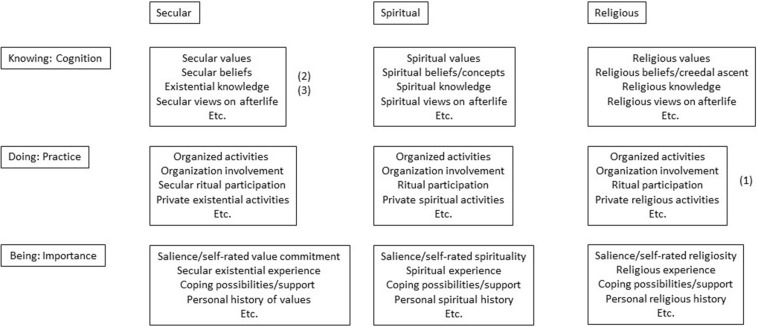
The Meaning-Making Matrix. Example: Through a “spiritual needs assessment” a patient is identified as a practicing catholic and is expressing a need to go to Mass, marked above by (1). The patient is also expressing a need to reconcile with an old friend and a need for talking with family about their financial situation, marked above by 2 and 3. This would lead to considering the following interventions: Organizing participation in a Mass, a visit by the old friend, and a visit by the family. However, all 3 interventions need to be reflected through the understanding of the patients ontological grounding and physical/mental condition: Is the patient physically capable of going to Mass and if not, what alternatives are there? Who is the friend and what is their history, is the patient in a mental condition to reach reconciliation or will such a meeting lead to further distress? Is the patient mentally capable of addressing financial issues and what are these issues, and will it lead to further distress? This leads to ethical considerations about how to approach the situation and who to involve; just as further interventions could be considered and leading to the development of the spiritual care treatment plan.

Where Figure 1 illustrates the ontological grounding of the patient, Figure 2, the Meaning-Making Matrix (MMM), inspired by la Cour and Hvidt (2010), sets this in relation to three central aspects of meaning-making: Knowing, Doing, and Being. The secular, spiritual, and religious components are here differentiated in relation to these aspects. The MMM enables the HCP to explicitly reflect upon the patient’s ontological grounding and what existential orientations the patient draws upon – and on that basis to locate what kind of interventions are needed to support coping through these orientations and to offer adequate and sensitive spiritual care. In this way the MMM facilitates the HCP in understanding the patient, thereby making it easier to address the area ethically, sensitively, and appropriately. The content of the boxes is similar to each other, only the frames “secular,” “spiritual,” “religious” change, in order to illustrate that the needs are, or may be, similar but that they are understood by the individual from different perspectives. Approaching the ontological grounding of the patient through the MMM will also enable an understanding of what aspects are important to the patient (relational, individual, embodied, verbal) (Viftrup et al., 2021), and finally, it will give an idea of who should be involved in providing spiritual care; nurse, physician, social worker, chaplain, relative, etc.

As will be exemplified below, this attempt at understanding the patient in relation to the MMM can be done in many ways, using questionnaires, through daily interactions and conversation, through the involvement of relatives or friends, etc. Essential is that the specificity of the needs is determined, i.e., whether they are of mainly secular, spiritual, or religious nature, whether they are of a cognitive, practical, or emotional kind, if they are interwoven, and how important various aspects are to the patient at various times. Can the needs be addressed through daily interaction and conversation with the nurse? Does the patient want to interact with peers, to have physical contact, listen to music? Or is the patient in such distress that a psychiatrist, psychologist, or chaplain is needed? Maybe it is a question of making peace with a higher being or participating in spiritual or religious rituals or other kinds of activities, in which case a chaplain or religious community may be needed. Maybe it is a combination of the above? Understanding the patient in relation to the MMM will assist in clarifying these questions and assist in identifying specific approaches relevant/suitable for the individual patient. In everyday practice this is likely to take place as the HCP is getting to know a patient as part of relationship-building. In this way the MMM is an explicit area of focus in the process of spiritual care, initiated when meeting the patient and an integrated part of the spiritual care treatment plan (phase 3 in the process of spiritual care).

We realize, that using the constructs “secular,” “spiritual,” and “religious” is already assuming “something” on behalf of the patient, namely that the ontological grounding and the MMM can be understood and accessed through these constructs. This is a bias and a limitation. However, by making these reflections explicit, we may reveal our potential bias and challenge our own understandings, and thereby reach a better understanding of the patient (Holbraad, 2017; Nissen et al., 2019b). Is this pushing the endeavor to far, is it complicating things more than need be? As we have argued, we find it necessary, in an individualized, culturally entwined, and pluralistic world, in order to gain an understanding of the patient that is recognizable to both patient and HCP, and from there to identify the appropriate actions, and develop the spiritual care treatment plan.

## The Process of Spiritual Care

In the following we will outline the process of spiritual care and then commence to a discussion of the process, exemplified by instruments/interventions focused on the different phases. The examples in the discussion have been drawn from the Catalogue of Spiritual Care Instruments (Damberg Nissen et al., 2020).

**FIGURE 3:**
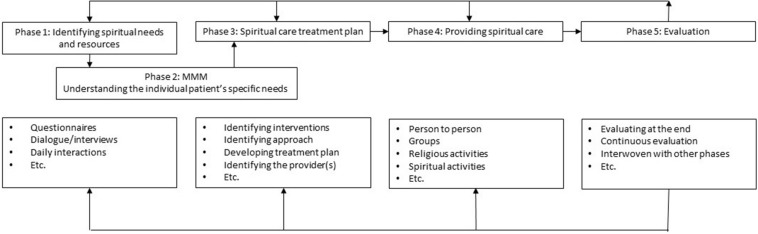
The Process of Spiritual Care.

Figure 3 illustrates the process of spiritual care as spanning 5 phases. The four boxes below the phases illustrate what kinds of instruments/interventions can be applied. The “etc.” has been included to illustrate that other possibilities exist.

The process of spiritual care is illustrated as starting by identifying spiritual needs. Phase 1 is then combined with phase 2 – the MMM, which leads to phase 3 – developing the spiritual care treatment plan and locating the relevant HCP’s to be involved. Phase 4 is then the actual provision of spiritual care. Phase 5 is the evaluation that should take place to ensure that the spiritual care provided is living up to expectations. The arrow going from “phase 5: Evaluation” and back to the previous phases illustrates that spiritual care should be continuously evaluated and the spiritual care treatment plan adjusted according to the findings of the evaluation.

## Examples and Discussion

### Phase 1: Identifying Spiritual Needs and Resources

In the following we have focused on the identification of spiritual needs. However, a similar approach can be taken in relation to spiritual resources. How are spiritual needs identified? They can be identified as part of the daily interaction between patient and HCP, through conversation or observation, this will be addressed below. First, we address the explicit assessment as an approach. The Catalogue of Spiritual Care Instruments locates 132 questionnaires within the field of spiritual care. Not all of these are spiritual needs assessment questionnaires but a recent overview, which was based on the Catalogue of Spiritual Care Instruments, included 22 questionnaires aimed at assessing spiritual needs or spiritual distress (Nissen and Hvidt, 2021). These spiritual needs assessment questionnaires were developed in different national and cultural contexts, reflecting the international attention on spiritual needs and spiritual care, but also reflecting local contextual differences in the way the questions are formulated. They span from containing strictly secular questions, such as “the Psychosocial and Spiritual Needs Evaluation scale” from Spain (Mateo-Ortega et al., 2019), “the Existential Distress Scale” from Canada (Lo et al., 2017), and “the Spirit 8” from South Africa and Uganda (Selman et al., 2012), to questionnaires that include explicit questions in relation to spirituality or religiosity, such as the “Geriatric Spiritual Well-being Scale” from the United States (Dunn, 2008), “the Holistic Health Status Questionnaire” from Hong Kong (Chan et al., 2016) and “the Spiritual Distress Scale” from Taiwan (Ku et al., 2010), while others have been developed in a religious setting such as the “Elder Spiritual Health Scale” from Iran (Ajamzibad et al., 2018), “the Spiritual Care Needs Scale” from Turkey (Otuzoglu, 2019) and “the Mature Religiosity Scale” from Netherlands (Vries-Schot and Uden, 2012). The “Thai Spiritual Well-being Assessment Tool for Elders with Chronic Illnesses” from Thailand (Unsanit et al., 2012), is an example of an assessment instrument developed in a pluralist religious setting (Buddhist, Islam, Christian) and containing no explicit references to religion (only one reference to Dharma). In the area of assessing spiritual needs and resources in the elderly, the Catalogue of Spiritual Care Instruments also mentions the “JAREL Spiritual Well-Being Scale” from the United States (Hungelmann et al., 1996) and the “Spiritual Distress Assessment Tool” from Switzerland (Monod et al., 2015).

“The Spiritual Needs Questionnaire” from Germany (Büssing et al., 2010) is likely to be the most widely distributed spiritual needs assessment instrument and has been translated, validated, and implemented in at least 18 countries (Damberg Nissen et al., 2020).

Spiritual needs may also surface in the day-to-day interaction between patient and HCP. As such, identifying spiritual needs becomes part of the relationship between patient, HCP, and other involved parties such as relatives and friends. From this perspective spiritual needs are not necessarily identified when the patient is diagnosed or hospitalized, but becomes part of relationship-building between patient and HCP (Steenfeldt, 2019), and thereby partly dependent on both the empathy of the HCP and the willingness of the HCP to engage in this work. To approach spiritual needs through daily interaction with the patient, underlines the need for spiritual care to be included in the curriculum, and the necessity of this has increasingly come into focus during the past decades. In an international context this also needs to be furthered by the concept “spirituality” to be continuously addressed and discussed in research, as the concept has so many different connotations and meanings, as an aspect of being human and as a concept in healthcare (Hvidt et al., 2020). “The Spiritual Assessment in Aging” from the United Kingdom (Nelson-Becker et al., 2007) was developed to both prepare and guide clinicians to undertake spiritual assessment through conversation, identifying 11 domains in spirituality through which a framework is assembled for spiritual assessment with older adults.

While identifying spiritual needs and resources through daily interactions is a sensitive and patient empowering approach, it demands great attention, preparation, and education/training on behalf of the HCP. In highly secular contexts this might be difficult, as there may be a tendency to marginalize spiritual needs, as both patient and HCP may find the topic difficult to engage, which is partly influenced by the surrounding (cultural) contexts tendency to marginalize the area as irrelevant, unimportant, or inappropriate. This hinders the identification of spiritual needs and resources, as has been documented in psychiatric research (Nissen et al., 2019a). Nissen (2019) outlines “the privacy of religion argument” as hindering spiritual needs from being identified in secular healthcare, because asking about spirituality or religiosity is considered so private that it becomes unethical to ask such questions directly. The patient must bring it up if spirituality/religiosity is to be brought into conversation. This is supported by the study on spiritual care in Danish hospices by Viftrup et al. (2021), and also by Andersen et al. (2020) study on existential communication between physicians and patients with chronic pain and multiple sclerosis. Being thus far a purely theoretical concept, it is unknown to what extent the MMM will assist in overcoming difficulties in relation to existential communication. However, the MMM will initiate a conscious reflection on behalf of the HCP in relation to the ontological grounding of the patient, which has the potential of therethrough making it easier to address the area ethically, sensitively, and appropriately, as it explicitly identifies spiritual needs and resources as relevant in the given (secular) healthcare context.

The United States Joint Commission on Accreditation of Healthcare Organization’s (JCAHO) has developed a set of guidelines to assist the HCP in assessing and identifying spiritual needs through day-to-day interaction. The guidelines are formulated as a series of questions for the HCP to be aware of when in contact with the patient. The patient is not necessarily asked these questions explicit but they enable the HCP to be aware of areas where spiritual needs might surface (Hodge, 2006). As such, the JCAHO guidelines is an example of a way to address phase 1 and 2 in the process of spiritual care, without presenting a stringent and predesigned approach to spiritual care. Wiltjer and Kendall argue that a spiritual needs assessment should be part of a holistic assessment of older people, where the spiritual domain is seen as one of five key domains (Wiltjer, 2019).

The nurse or social worker is likely to be in a good position to both identify spiritual needs and resources and provide spiritual care, considering that relationship is a central part of providing spiritual care. This is underlined by spiritual distress having been a diagnose in nursing in the United States since 1978 (Hodge, 2006), and as Burkhart et al. (2011) argues, spiritual care has always been an integral part of nursing care.

The general practitioner, who is often the HCP who often knows the patient prior to severe illness, is also in a position to identify spiritual needs and resources, but is often not in a position to offer spiritual care, simply because of time limitation in general practice (Assing Hvidt et al., 2017a). This does not mean that spiritual care cannot be part of general practice. The “existential communication in general practice” tool (EMAP) from Denmark, is an example of an instrument facilitating communication about existential needs and resources between general practitioners and patients with cancer (Assing Hvidt et al., 2017b), also functioning as a way for the general practitioner to open the topic, without transgressing ethical or personal borders, thereby overcoming the above-mentioned “privacy of religion argument” and facilitating working with the MMM.

The chaplain is trained and experienced in having existential conversations and in providing spiritual care. With the ambition of sharing this expertise with HCPs’, Fitchett and Risk (2009) from the United States developed the “Religious Struggle Screening Protocol” (RSSP). The RSSP was developed in a Christian context within chaplaincy, with the intention of assisting non-chaplain HCP’s to identify patients in need of spiritual care. It is a map of action consisting of a series of yes/no questions for the HCP to be attentive of when talking with the patient leading to different action outcomes. In the secular countries of Scandinavia and Northern Europe, the chaplain is experienced in interfaith dialog and is able to provide spiritual care in different frameworks and address secular, spiritual, and religious existential question alike (Nissen et al., 2019b).

### Phase 2: The Meaning-Making Matrix

Once spiritual needs have been identified, it is necessary to locate the nature of the spiritual needs. Involving the MMM will help to clarify whether the identified needs are of a secular, spiritual, or religious kind, whether there are cultural variances that need be taken into consideration, and whether the needs are of a cognitive or practical nature, or a combination of this, as outlined above in section “The Ontological Grounding and the Meaning-Making Matrix”.

The “Cultural Formulation Interview” (CFI) from the United States (Aggarwal et al., 2014) is an example of an approach that can assist in understanding the patient through the MMM. The CFI is not aimed at spiritual care *per se*, but it contains (culturally) open questions that will enable the patient to reflect on the personal background and context, and therethrough the HCP will be able to get an understanding of the patient in relation to the MMM. The “ETHNIC(S) mnemonic” from the United States (Kobylarz et al., 2002) is another example in relation to the MMM. The “ETHNIC(S) mnemonic” was developed in Geriatric care as a framework that practitioners can use in providing culturally appropriate care for the elderly.

As illustrated in Figure 3 we see the MMM as interwoven in phases 1–3, meaning to illustrate that understanding the individual patients ontological grounding is essential for addressing the questions of how to care for the patient in relation to the identified spiritual needs, what are the specifics of these needs, what are the relevant interventions, and who are the relevant HCP’s to be involved? These are explicit reflections to be made in relation to the individual patient and will influence the HCPs to be involved in both identifying spiritual needs (phase 1), developing the spiritual care treatment plan (phase 3), and who should be involved in providing spiritual care (phase 4).

### Phase 3: The Spiritual Care Treatment Plan

Having identified spiritual needs and reached an understanding of these needs in relation to the patients ontological grounding, should enable a point from which to develop a plan for the provision of the spiritual care. Who is qualified to develop this plan? A chaplain, a general practitioner, a psychologist, a nurse, a relative, or is it a joint effort? We propose that this is a joint effort, as spiritual care is best practiced as a teamwork effort, and as part of holistic and patient centered healthcare it could potentially involve all concerned parties, as an interprofessional endeavor (Puchalski et al., 2006, 2019; Bandini et al., 2018). The particular spiritual care treatment plan will reflect the patient in relation to the ontological grounding and whether the nature of the identified spiritual needs is of a secular, spiritual, or religious character, and whether the needs are of a cognitive, practical, or emotional kind, or a mix. The spiritual care treatment plan should reflect the interventions included and how the actual provision of spiritual care should be implemented; who should be involved to do what, when, and where? The “Spiritual Assessment and Intervention Model” (Spiritual Aim) from the United States (Shields et al., 2015) is an example of such an approach. Shields and colleagues argue that if spiritual care is provided without a plan, then the intervention(s) may stray off course, or simply remain within the realm of a social visit or random interactions with patients. While Spiritual Aim is not a spiritual care treatment plane in itself, it could inspire as a conceptual framework for the development of specific spiritual care treatment plans as it can assist the HCP to diagnose a patient’s unmet spiritual needs (phase 1/2), to devise a spiritual care treatment plan (phase 3), to implement this plan (phase 4), and to evaluate the desired and actual outcome of the intervention (phase 5). As such, Spiritual Aim is an example of an approach encompassing all phases. Spiritual Aim was developed from a Lutheran perspective but is now inclusive of other faiths and implemented in clinical settings. Its applicability in secular context or with secular oriented patients is a question for further research. Another example of a systematic approach that includes all phases of assessing, planning, providing, and evaluating spiritual care is “Guidelines for the Assessment of Spiritual Needs” from the United Kingdom (Govier, 2000).

### Phase 4: Providing Spiritual Care

Providing spiritual care is implementing the spiritual care treatment plan. Interestingly, even though many spiritual care instruments exist, approaches for providing spiritual care through the provision of a spiritual care treatment plan seem scarce (Harrad et al., 2019; Damberg Nissen et al., 2020). This might be because, as we have argued, spiritual care is an individual and relational process and therefore difficult to put into stringent formulae; it must be developed at the local level with the individual patient in mind. “Spiritual Reminiscence” from Australia (Mackinlay and Trevitt, 2010) is an example of an approach that both enables identifying spiritual needs (phase 1) while also being applicable as part of the provision of spiritual care, in the sense that spiritual reminiscence is a type of narrative gerontology, enabling the elderly to give meaning to their life-story while also connecting socially to peers.

### Phase 5: Evaluation

Phase 5, evaluation, should be included as part of the spiritual care treatment plan and take place continuously in order to secure that the care is being provided according to plan, and that effect be measured ongoingly in order to adjust the spiritual care treatment plan if necessary. Evaluating a process can be done in many ways but should be integratable with the identification/assessment of spiritual needs to enable effect evaluation. If this kind of evaluation is intended, it will be made possible by identifying/assessing the spiritual needs through a questionnaire and then reusing the questionnaire for evaluative purposes, in view of gaging to what extent the needs have been met. There are instruments made to specifically assess the effect of an intervention, such as the Service-user Recovery Evaluation Scale (SeRvE) from England, which is a patient reported outcome measure developed to monitor interventions, which also highlights the importance of spiritual care for patients (Barber et al., 2018).

## Perspectives and Conclusion

To approach spiritual care as a process has implications and perspectives for all healthcare areas where existential questions and crises may arise, be it in geriatrics for an elderly at the end of life, in pediatrics for a youngster diagnosed with cancer, or anyone in between faced with the difficulties of existential crisis. It also has consequences for the person(s) who provide spiritual care, be it the general practitioner, the nurse, the psychologist, the chaplain, the relatives, or anyone else who cares for persons in existential crisis. We have argued that it is difficult to develop a stringent approach that encapsulates the process of spiritual care as a whole, because spiritual care is a relational and individual process that takes place in a local context between individuals, and therefore each case of providing spiritual care is unique. By drawing on examples from the Catalogue of Spiritual Care Instruments we have illustrated that many instruments exist that in one way or another are applicable in the process of spiritual care, but also that they cannot stand alone and that several instruments are necessary. This points to the conclusion that developing a spiritual care treatment plan is essential for providing spiritual care, so that spiritual care does not become *ad hoc* solutions depending on individual empathy, involvement, (lack of) training, and interest.

We have illustrated the importance of locating patients in relation to their ontological grounding and from there to approach an understanding of the patient in relation to secular, spiritual and/or religious existential needs. We have argued this as essential in a culturally entwined and pluralist world, and we have suggested the ontological grounding by means of the MMM as a way to approach this aspect, as it will help to understand the specificity of the individual patient’s spiritual needs and thereby assist in developing the best possible spiritual care treatment plan. How this spiritual care treatment plan should be developed in various international and vernacular contexts is an area that calls for further attention and research, to delineate and share international experiences and best practice. Our presentation of the ontological grounding and MMM should also assist in this aspect of the international development of spiritual care, as it is not specific to culture, spirituality, or religion, nor does it present an intervention, but a structure in which to implement specific instruments/interventions.

Presenting spiritual care as a process implies working within a conceptually defined spiritual care provision framework. We recommend this to be the default position for any institution where spiritual care is part of the daily work and routines. This so, especially because looking at spiritual care as a process highlights that moving from identifying spiritual needs in a patient, to the actual provision of spiritual care involves deliberate and considered actions that consider the patient’s specific ontological grounding. This also implies the need to identify the appropriate personnel to provide spiritual care and assist the involved parties in developing the best possible spiritual care treatment plan.

By presenting spiritual care as a process, we hope to inspire the focus on spiritual care as a whole, as a relationship between the involved parties, as a way to make visible the necessities, the difficulties but, most importantly, the positive potential that lies in spiritual care. In the final instance, spiritual care has only one ambition; to help the individual human being through crisis.

## Data Availability Statement

The original contributions presented in the study are included in the article/supplementary material, further inquiries can be directed to the corresponding author.

## Author Contributions

DTV and NCH discussed the topic and reviewed the manuscript. All authors contributed to the article and approved the submitted version.

## Conflict of Interest

The authors declare that the research was conducted in the absence of any commercial or financial relationships that could be construed as a potential conflict of interest. The authors declare that this study received funding from Pfizer Oncology Denmark. The funder was not involved in the study design, collection, analysis, interpretation of data, the writing of this article or the decision to submit it for publication.

## Publisher’s Note

All claims expressed in this article are solely those of the authors and do not necessarily represent those of their affiliated organizations, or those of the publisher, the editors and the reviewers. Any product that may be evaluated in this article, or claim that may be made by its manufacturer, is not guaranteed or endorsed by the publisher.
